# Deciphering the multidrug resistance paradigm in *Candida auris*

**DOI:** 10.1128/aac.01062-24

**Published:** 2026-03-16

**Authors:** Darian J. Santana, Nicholas C. Cauldron, P. David Rogers, Christina A. Cuomo

**Affiliations:** 1 Department of Pharmacy and Pharmaceutical Sciences, St. Jude Children’s Research Hospital 5417, Memphis, Tennessee, USA; 2 Department of Molecular Microbiology and Immunology, Brown University 6752, Providence, Rhode Island, USA; University of Iowa, Iowa City, Iowa, USA

**Keywords:** *Candida auris*, drug resistance, fungal genetics

## Abstract

*Candida auris* has garnered substantial clinical and public health attention for its widespread antifungal resistance. Most isolates are resistant to fluconazole, and many, to other drug classes, with acquired resistance to all clinically available antifungal drugs reported. Antifungal resistance is rising alongside increasing case counts, threatening a sparse antifungal toolbox with multidrug and pan-resistant isolates that may cause untreatable infections. In this minireview, we examine the recent literature investigating the mechanisms and evolutionary patterns of resistance in clinical isolates of *C. auris* to each antifungal utilized to combat these infections. We propose a refined model of *C. auris* drug resistance by separating the multidrug resistance paradigm into distinct resistance challenges for each drug class. We examine how the emergence of unique resistance patterns to each drug may suggest therapeutic options even for currently available antifungals. Resistance to fluconazole is driven by drug target mutations with clade-specific representation and more diverse acquired mutations in drug efflux regulators. Recent structural insights into the context of these mutations may suggest vulnerabilities to other triazoles even in fluconazole-resistant strains. Acquired resistance to echinocandins, amphotericin B, and the pyrimidine analog flucytosine is rare but can emerge under antifungal therapy through conserved resistance mechanisms. The reportedly higher amphotericin B resistance rate in *C. auris* relative to other *Candida* species remains poorly understood and may be linked to unexplored intrinsic resistance mechanisms. We suggest that close examination and further investigation of these mechanisms may inform better therapeutic practice and may offer treatment solutions for this multidrug-resistant pathogen.

## INTRODUCTION

*Candida auris* (taxonomically reclassified as *Candidozyma auris* [1]) is often described as a paragon of antifungal resistance among fungal species. Like the related *Candida haemulonii* (reclassified as *Candidozyma haemuli*) complex members, *C. auris* demonstrates a notably high propensity for antifungal resistance compared to more commonly clinically isolated but genetically distant *Candida* species. As *C. auris* has emerged and become more commonly established, concerns around diminishing therapeutic options in an already limited arsenal of antifungal drugs have prompted substantial warnings from clinical and public health authorities (2–4). Our understanding of *C. auris* antifungal resistance is grounded in early studies that characterized the global spread of this pathogen (5, 6). While the numbers vary across regional reports, nearly 90% of clinical isolates are resistant to at least fluconazole, a widely administered triazole-class drug, while up to 30% of isolates are reportedly also resistant to a drug in at least one other antifungal class. Research in subsequent years has deeply investigated mechanisms and patterns in *C. auris* antifungal resistance, presenting the opportunity for reexamination to identify therapeutically useful insights (7–9).

Surveillance, sampling, and genome sequencing data have enabled the inference of drug resistance mutations and their evolution in *C. auris* populations (5, 6, 10, 11). In parallel, studies tracing the evolution of resistance in sequential isolates, both *in vitro* and in patients, have similarly implicated resistance mechanisms (11–17). In turn, experimental validation has detailed the impact of resistance contributions, with surveillance data tracing the occurrence and expansion of the impactful mutations through lineages.

In this minireview, we summarize how this combination of genomic, experimental, and clinical comparative approaches has advanced our understanding of the mechanisms underlying *C. auris* resistance to drugs in all three major classes of antifungals available for therapeutic use—azoles, echinocandins, and polyenes—and flucytosine, a pyrimidine analog. We then integrate this mechanistic knowledge to examine new resistance emergence in clinical settings, explore where mechanistic details may inform reevaluation of therapeutic options, and offer perspectives around the current dynamics and future trajectory of resistance in *C. auris*.

## AZOLE RESISTANCE

**Fig 1:**
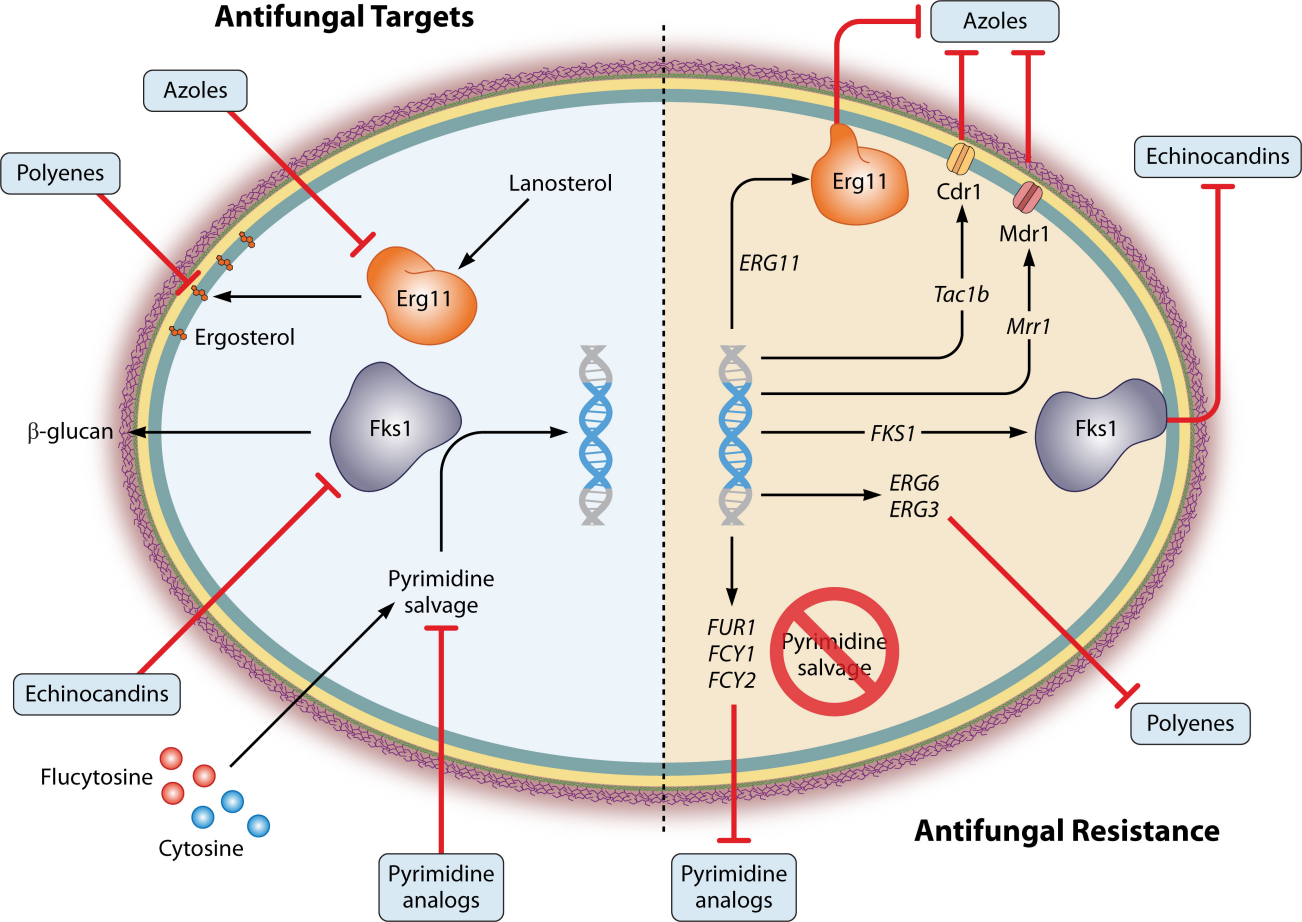
Antifungal targets and mechanisms of acquired antifungal resistance. The triazoles and echinocandins target fungal enzymes Erg11 and Fks1, respectively. Mutations in these drug targets promote resistance. Mutations leading to the upregulation of efflux pumps also contribute to triazole resistance. Antifungal activity driven by polyenes is driven by binding ergosterol in the cell membrane. Mutations in ergosterol biosynthetic pathway genes such as *ERG6* and *ERG3* limit ergosterol accumulation in the membrane, rendering polyenes inactive against resistant mutants. Pyrimidine analogs, such as flucytosine, are metabolized into nucleotoxic downstream analogs. Loss of function of any of the genes responsible for this metabolism, such as *FUR1*, *FCY1*, or *FCY2*, ablates the antifungal activity of these compounds.

The extremely high rate of fluconazole resistance in *C. auris* sets this species apart from other pathogenic fungi, where lower rates are observed. *C. auris* resistant isolates also commonly demonstrate resistance in very high fluconazole concentrations, well above resistance breakpoints used for other species. These patterns are explained by complex combinations of acquired resistance mutations (Fig. 1). Here, we detail the intersection of these mutations with mechanisms of drug activity.

### Drug background and mode of action

**Fig 2:**
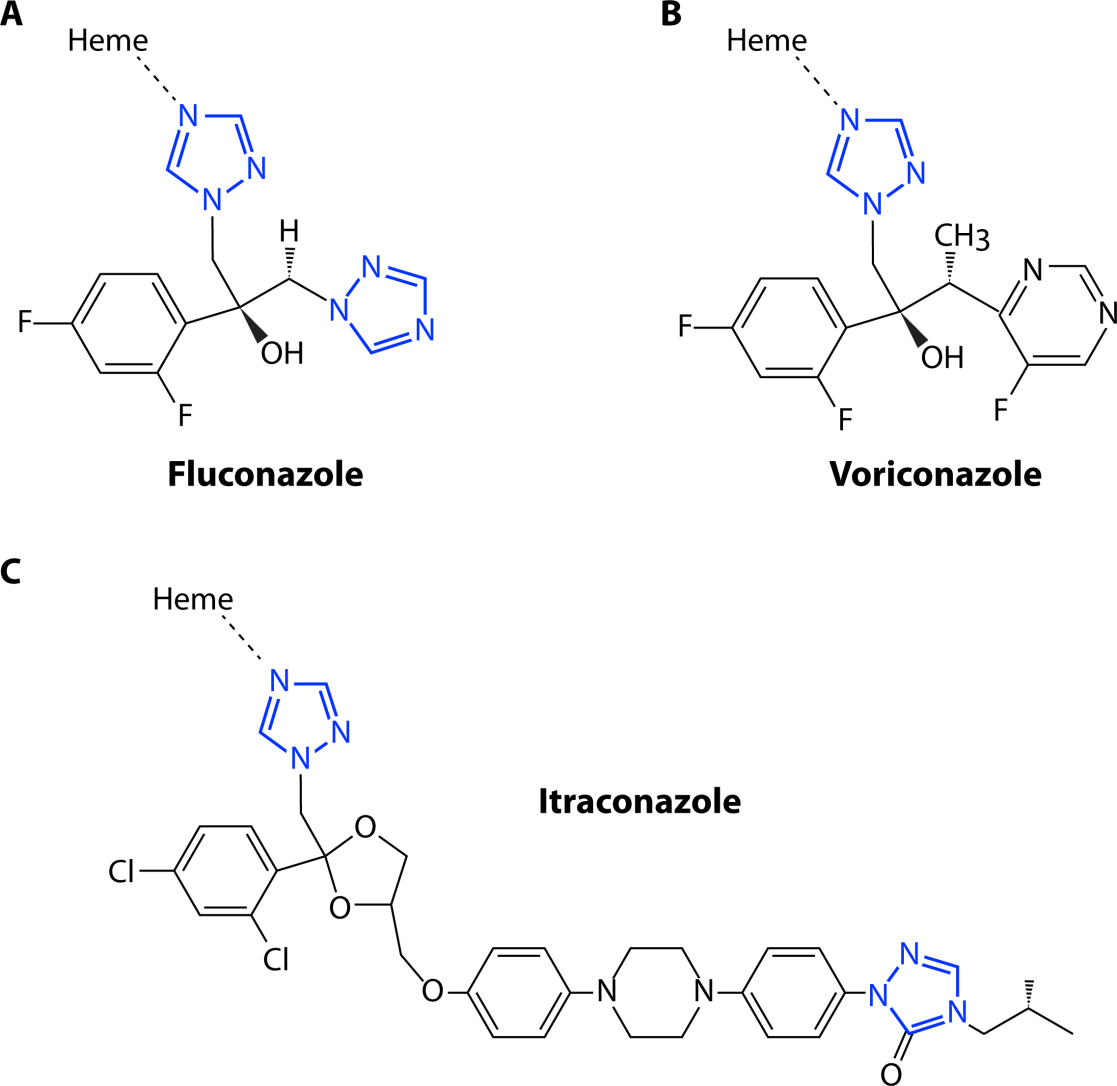
Chemical structures of representative triazoles. (**A**) Fluconazole: The N4 nitrogen of one of the two triazole rings (blue) is coordinated to the iron within the heme cofactor in the Erg11 active site. The hydroxyl group and N4 of the second triazole ring participate in hydrogen bond networks with amino acids at the active site. The variability in modifications and structures of the second triazole ring in different drug compounds influences such interactions with the Erg11 protein, while at least one 1,2,4-triazole ring coordinates to the heme. (**B**) Voriconazole: Similar to Fluconazole except that the second triazole ring is replaced with a fluoropyrimidine group and a methyl group. This change may shift the orientation of the molecule within the hydrogen bonding networks. (**C**) Itraconazole: “Long-chain” triazoles such as Itraconazole are modified with a long nonpolar side chain that facilitates Van der Waals and hydrophobic interactions with the apoprotein of the Erg11 enzyme.

The azoles are synthetic antifungals that inhibit the biosynthesis of ergosterol, the main sterol component of fungal plasma membranes. Their activity is primarily fungistatic against *Candida* species (18, 19). Azole drugs consist of a heterocyclic ring, containing either two nitrogens (in the case of the first-generation imidazoles) or three nitrogens (in the later-developed triazoles), and a variable side chain (Fig. 2). The drug target cytochrome P450 enzyme relies on the coordination of a heme cofactor at the active site to catalyze the rate-limiting lanosterol 14α-demethylase step during ergosterol biosynthesis (20). The nitrogen molecules of the azole rings directly interact with the heme ferric ion, while the side chains interact with amino acid residues in the enzyme active site (21, 22). This competitive binding inhibits demethylation of the endogenous lanosterol substrate (23), limiting the generation of ergosterol, essential for membrane fluidity and permeability (20, 24, 25). It has also been widely proposed that disruption of the ergosterol biosynthetic pathway results in the accumulation of pathway intermediates, which may themselves become toxic when incorporated in the membrane (26). This model is primarily supported by observations that mutations upstream in this biosynthetic pathway can alter the pool of accumulated intermediates and confer indirect azole resistance (27).

In *Candida* species, the *ERG11* gene encodes the azole target cytochrome P450 enzyme (referred to here as Erg11). A human homolog of this enzyme exists (28), leading to cross-reactivity among early imidazole drugs (29, 30). The subsequent development of the triazoles sought to overcome many of the shortcomings of their imidazole predecessors, leading to widespread antifungal utility (31). Among the triazoles, fluconazole is recommended most frequently as primary or step-down therapy for many types of *Candida* infection due to its low cost, favorable toxicity, pharmacokinetic profile, and availability as both intravenous and oral formulations. Resistance among most *Candida* species remains rare. Less than 1% of *C. albicans* isolates harbor fluconazole resistance (32, 33). About 10% of global *Nakaseomyces glabratus* collections can be resistant to fluconazole, and rates up to 30% have been reported (32–35).

Fluconazole resistance, in particular, sets *C. auris* apart from other *Candida* species. It is estimated that 80%–90% of *C. auris* isolates exhibit fluconazole resistance, and microbiological resistance can reach very high levels (5, 36). For most other *Candida* species, a fluconazole MIC (minimum inhibitory concentration) approaching or exceeding 64 μg/mL is exceptionally rare among wild-type isolates (37). For *C. auris*, MICs exceeding that level by 4-fold or even 8-fold are commonplace. Because of the frequency of resistance, fluconazole currently has limited utility for *C. auris*.

The CDC guidance on antifungal susceptibility testing notes that isolates resistant to fluconazole may respond to other triazoles. Voriconazole, for instance, has shown some clinical use as step-down therapy for fluconazole-resistant *Candida krusei* and *N. glabratus* (38), and similar trends may emerge for *C. auris*. Other triazoles have weaker recommendations than fluconazole and voriconazole in clinical practice guidelines for the treatment of candidiasis but may be options for consideration when resistance limits viable alternatives (38, 39).

### Mechanisms of resistance: ERG11 mutation

Mutations in the drug target *ERG11* gene itself are commonly associated with fluconazole resistance in *C. auris*. There are three predominant mutations among global isolates with distinctly clade-specific representation: the two major subclades of Clade I isolates encode, respectively, Erg11 K143R or Y132F, nearly all Clade III isolates encode the F126L resistance mutation along with the adjacent V125A mutation, and most resistant Clade IV isolates encode Y132F (Table 1) (6, 16, 40). The subclades of isolates harboring these mutations are estimated to have emerged as recently as the past several decades (5). While their relation to susceptible strains and strains with wild-type *ERG11* suggests that *C. auris* is not intrinsically resistant to fluconazole, many outbreaks are caused by these triazole-resistant subclades (4, 41, 42). These *ERG11* alleles are largely fixed across large proportions of isolates of each clade, although some mutations can rarely be observed appearing independently of clade, such as Clade IV isolates harboring the K143R primarily found in Clade Ic (16, 43). This supports growing evidence of parallel and ongoing evolution of triazole resistance across clades.

**TABLE 1:** Mutations observed in clinical isolates linked to decreased drug sensitivities

Drug class	Gene	*C. auris* variants^*a*^	Clades represented
Azoles	*ERG11* – Lanosterol 14-a-demethylase	**Y132F**	I and IV
		**K143R**	I and IV
		**F126L**	III
	*TAC1B* – Zinc finger transcription factor	**A640V**	I
		**A657V**	I
		**F862_N866del**	IV
	*MRR1A* – Zinc finger transcription factor	**N647T**	III
Polyenes	*ERG6* – C-24 sterol methyltransferase	**R97fs**	I
	*ERG3* – C-5 sterol desaturase	**G71fs**	III
Echinocandins	*FKS1* - β-1,3-glucan synthase	**S639F,** S639Y	I and III
		S639P	I and IV
		Δ635F, F635C, F635Y, F635L, D642Y, M690V, **W691L**, R1354S, **R1354H**	I

^*a*^ Variants in bold have experimental validation showing elevation of MIC values above the relevant resistance breakpoint.

Acquisition of any one of three *ERG11* mutations (V125A, Y132F, and K143R) drives azole resistance in *C. auris*. Nearly all isolates harboring one of these mutations are microbiologically resistant to fluconazole (MIC ≥ 32 µg/mL) (5). One early report heterologously expressed *ERG11* alleles harboring these mutations in *Saccharomyces cerevisiae*, increasing fluconazole MIC values 4-fold to 8-fold (44). Another experiment replaced the wild-type *ERG11* allele in a fluconazole-susceptible *C. auris* isolate with each variant allele, leading to up to a 16-fold increase in fluconazole MIC (45). In the same study, replacement of the K143R mutation with the fully functional *ERG11* allele resulted in an 8-fold decrease in fluconazole MIC. Notably, these shifts in MIC were not always sufficient to cross the clinical breakpoint, demonstrating the requirement of multiple resistance mechanisms to reach high-level resistance. Each of these mutations also showed smaller shifts in sensitivity to voriconazole, affecting MICs by 2-fold to 4-fold, while negligibly influencing MICs to isavuconazole, itraconazole, and posaconazole. A small percentage of isolates are thought to harbor multiple copies of *ERG11*, which may compound the impact of these mutations on resistance levels (5).

**Fig 3:**
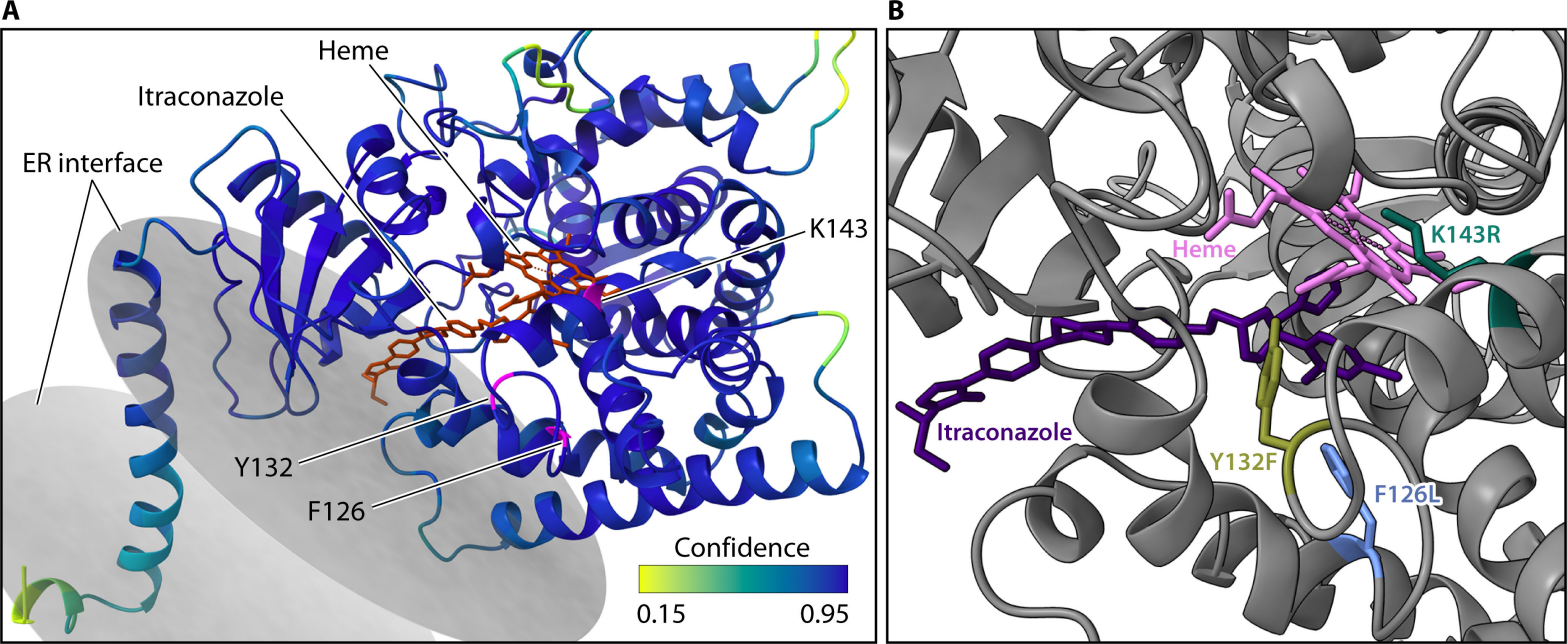
Three Erg11 mutations shown directly to elevate triazole resistance in *C. auris* are located near and face inward toward the enzymatically active site. Three-dimensional structure was modeled with the SWISS-MODEL homology-modeling pipeline using a cryo-electron microscopy structure of *S. cerevisiae* Erg11 bound with itraconazole and heme ligands (*chimerax 1.8* [48]; PDB 5EQB). (**A**) Model confidence (*QMEANDisCo* [49]) was high near three mutations that induce triazole resistance. The mutations are colored magenta and are labeled by their wild-type amino acid and position in the primary sequence. A small portion of Erg11 is embedded in the endoplasmic reticulum, and the membrane contacts are shown as disks (*PPM 3.0* [50]). (**B**) The three mutations are shown as the wild-type allele and labeled with the variant observed in *C. auris* populations. K143R and Y132F likely impact the protein’s ability to bind azoles, such as itraconazole (purple), or impact the redox potential of the heme ferric ion, shown in pink and with dotted lines toward the interacting heme.

Based on crystal structures in homologous Erg11 proteins from *S. cerevisiae* and *C. albicans*, each of the three most common *C. auris* Erg11 resistance mutations—Y132F, K143R, and F126L—is encoded near the enzyme active site (Fig. 3). Homologous residues to Y132 and F126 reside within 4 Å of a fluconazole molecule crystallized with *S. cerevisiae* Erg11, while the residue homologous to K143 likely interacts with the heme at the active site (46). The tyrosine at position 132 appears to maintain a critical hydrogen bonding network between a hydroxyl group on the azole and a propionate group on the heme (46). Mutation of this residue to phenylalanine would abolish this hydrogen bond, likely affecting the coordination between the azole nitrogen group and the heme molecule. The lysine at position 143 is located on the opposite face of the heme and interacts with the same heme propionate (46, 47). The heme is coordinated between K143 and Y132, and mutation of either is likely to impact the redox potential of the heme iron or its ability to bind azoles in the catalytic pocket. The direct mechanistic role of F126 in coordinating fluconazole at the active site is less clear than that proposed for the other residues, although its proximity to the drug in crystal structures likely contributes to a permissive binding environment.

The enzymatic impacts of mutations at these residues have been assessed in *C. albicans*, but not yet in *C. auris*. One study generated recombinant *C. albicans* Erg11 mutations harboring different resistance mutations and measured the enzymatic turnover, fluconazole binding, and enzymatic IC50 at varying fluconazole doses for purified enzymes (51). In this investigation, K143R, and to a lesser extent, Y132F mutations resulted in increased residual catalytic activity under fluconazole inhibition and greater fluconazole IC50 values compared to the wild-type enzyme. The K143R mutation displayed similar effects for voriconazole, itraconazole, and posaconazole, while the Y132F mutation specifically impacted only interaction with fluconazole. This study also assessed a mutation in the phenylalanine at position 126, although the authors used the valine substitution commonly found in *C. albicans* (F126V), and this substitution did not influence the ability of triazoles to inhibit the catalytic activity of the purified enzyme. Since each of the three predominant mutations to *C. auris ERG11* results in a similar magnitude of triazole MIC shifts, the relationship between structural or molecular differences in these key resistance residues and their impact on resistance is unclear (45).

While other less common *ERG11* variants have been identified in clinical isolates, few have been experimentally connected to resistance. A cluster of fluconazole-resistant Clade IV isolates from Colombia encoded an F444L mutation in *ERG11*, and expression of this allele in a susceptible background resulted in a 4-fold increase in MIC to both fluconazole and voriconazole (52). In contrast, two other mutations found in resistant *C. auris* isolates—I466M and Y501H—had no influence on fluconazole or voriconazole MIC when expressed in *S. cerevisiae* (53). These mutations lie outside the enzyme active site, and their mechanisms of conferring resistance are less well understood, although there are examples in *C. albicans* of substitutions on the enzyme proximal surface or external loops conferring triazole resistance (54, 55).

### Mechanisms of resistance: efflux pump regulation

Proteins of the ATP-binding cassette (ABC) and major facilitator superfamily (MFS) transporter families are broadly associated with triazole resistance in *Candida* species (56). Given the roles of these proteins in facilitating the movement of solutes across cell membranes, the most common models suggest transporter overexpression confers resistance by limiting intracellular drug concentrations. Among putative efflux pumps encoded by *C. auris*, the ABC transporter, Cdr1, and the MFS transporter, Mdr1, have primarily been implicated in triazole resistance.

The Cdr1 transporter appears to play a central role in *C. auris* fluconazole responses, and the regulation of its expression enables high-level resistance. The deletion of Cdr1 in a highly resistant background resulted in a 128-fold decrease in fluconazole MIC (57). Basal *CDR1* expression varies among *C. auris* isolates but is generally correlated with fluconazole susceptibility (57, 58). In *C. albicans*, *CDR1* transcription is regulated in part by the zinc-finger transcription factor Tac1 (Transcriptional Activator of *CDR*), which binds to drug-responsive elements in the promoters of *CDR1* and other transporters to mediate upregulation (59, 60). Mutations in *TAC1* can result in hyperactivation or constitutive expression of *CDR1* even in the absence of stimuli and are often associated with fluconazole resistance (56). *C. auris* encodes two putative homologs*—TAC1a* and *TAC1b*—which are encoded in tandem but share only approximately 25% sequence identity with one another (61). Notably, mutations in only *TAC1b* have been repeatedly identified in fluconazole-resistant isolates from *in vitro* evolution experiments (13, 14, 16, 62) and patient isolates (16, 17, 63, 64).

Of the more than a dozen *TAC1b* mutations reported in resistant clinical isolates, three common mutations—A640V, A657V, and F862_N866del—are exclusively encoded by highly resistant isolates and show frequent representation among isolates from Clade I and Clade IV (Table 1) (16). Expression of any of these alleles in susceptible *C. auris* strains results in constitutive upregulation of *CDR1* expression and increased MICs to fluconazole, itraconazole, posaconazole, voriconazole, and isavuconazole (65). Notably, each mutation controls a distinct regulon: each of the three mutations leads to upregulation of *CDR1* and *MDR1* and the dysregulation of other predicted transporter genes, with some variability in the size of the associated regulon (65).

Cdr1, and to a lesser extent Mdr1, are the major drivers of azole resistance associated with *TAC1b* mutations. Deletion of *CDR1* largely abrogates the increased triazole MIC associated with each *TAC1b* mutation, while deletion of *MDR1* in the absence of *CDR1* further reduces the MIC to fluconazole and voriconazole (65). The three predominant *TAC1b* mutations in *C. auris* affect constitutive *CDR1* expression, but this effect appears to be independent of drug-induced expression: when exposed to fluconazole, strains harboring *TAC1b* mutations exhibit multiplicative fold increases in *CDR1* (65). Drug-responsive *CDR1* induction in *C. auris* appears to only occur under supra-inhibitory concentrations of fluconazole, suggesting the constitutive basal increase in *CDR1* expression is the more likely driver of increased MICs (65). In *C. albicans*, wild-type *TAC1* is also critical for maintaining a basal level of *CDR1* expression even in susceptible isolates, but this relationship may not be conserved in *C. auris,* as deletion of *TAC1b* or both *TAC1a* and *TAC1b* does not result in decreased efflux pump expression (52, 61). Alteration of other regulatory pathways upstream of *CDR1* can also lead to resistance, demonstrated by mutation of a regulatory network including Ubr2, Mub1, and Rpn4 present in many Clade I isolates (66, 67).

The potential for multiple avenues of *CDR1* upregulation, and the extent to which associated mutations are apparently maintained in circulating *C. auris* populations, is concerning for multiple reasons. For one, as discussed above, *CDR1* expression can apparently influence not only resistance to fluconazole but all clinically available triazoles. Second, triazole resistance driven by *CDR1* is additive with that driven by *ERG11* mutation, and many isolates with very high MICs exhibit both resistance mechanisms (16). Third, the pleiotropic efflux activity of *CDR1* potentially threatens the efficacy of novel therapies. For instance, *TAC1b* mutations reduce the susceptibility of *C. auris* to the investigational drug manogepix in a *CDR1*-dependent fashion (68, 69). Two independent analyses found correlations between fluconazole resistance and increased manogepix MICs among collections of hundreds of *C. auris* isolates (70, 71).

In contrast to *CDR1*, *MDR1* appears to play a minor role in *C. auris* triazole resistance. Deletion of *MDR1* or the gene encoding its major transcriptional regulator *MRR1a* negligibly affects *C. auris* fluconazole resistance (57, 61, 65). Its influence may be masked by *CDR1*, since the loss of *MDR1* does demonstrate a modest effect on MICs in the absence of *CDR1* (57, 61, 65). The N647T mutation in *MRR1* is present in most Clade III resistant isolates concomitant with the F126L *ERG11* mutation (72). This mutation accounts for a roughly 6-fold increase in expression of *MDR1* and a modest shift in fluconazole or voriconazole MIC but does not influence resistance to the other triazoles (72, 73). Nonetheless, even this modest shift highlights the paradigm of fluconazole resistance in *C. auris*, where most resistant isolates encode multiple additive resistance mechanisms.

## ECHINOCANDIN RESISTANCE

In contrast to triazole resistance, echinocandin resistance in *C. auris* remains rare, represented in less than 10% of isolates (4–6, 74). Even this level of resistance represents great concern, however, as echinocandins are the most strongly recommended option to treat *C. auris* (38, 39). Resistance can emerge upon treatment, primarily driven by mutation of the drug target gene, and subsequent resistant isolates have contributed to outbreaks (75).

### Drug background and mode of action

The echinocandins exhibit fungicidal activity against most *Candida* species (76, 77) but are likely fungistatic against *C. auris* (78). They noncompetitively bind to the Fks1 subunit of the transmembrane heteromeric glycosyltransferase β-(1,3)-D-glucan synthase to block the production of β-(1,3)-D-glucan, a critical structural component of the fungal cell wall and the main scaffold on which cell wall proteins are anchored (Fig. 1) (79–82).

The clinically used echinocandins are built on the chemical backbone of naturally occurring antimycotic fungal metabolites (83). These compounds share a core cyclic hexapeptide acylated with a fatty acid that anchors the compound in the fungal membrane (Fig. 3) (84, 85). Semi-synthetic modifications were made to the core compounds to address hemolytic activity, poor solubility, and host toxicity, resulting in the near-concurrent introduction of caspofungin, micafungin, and anidulafungin as antimycotic therapies (86).

Because they target a fungal-specific process, echinocandins are selective and well-tolerated as therapeutics. The primary limitation of echinocandin therapy is the requirement for daily parenteral administration, although this is slightly alleviated by the most modern echinocandin, rezafungin, which is more stable to reduce dosage frequency (38, 83, 87). Otherwise, all echinocandins have minimal adverse effects, and pharmacologic properties are similar (38). Likely for these reasons, the echinocandins have performed well in clinical trials and are recommended as first-line therapy for adult patients with invasive candidemia, and this recommendation extends to *C. auris* (38, 39). Where the applicability of triazoles as an alternative is limited by the high rates of triazole resistance, echinocandins are particularly essential for the management of *C. auris*.

**Fig 4:**
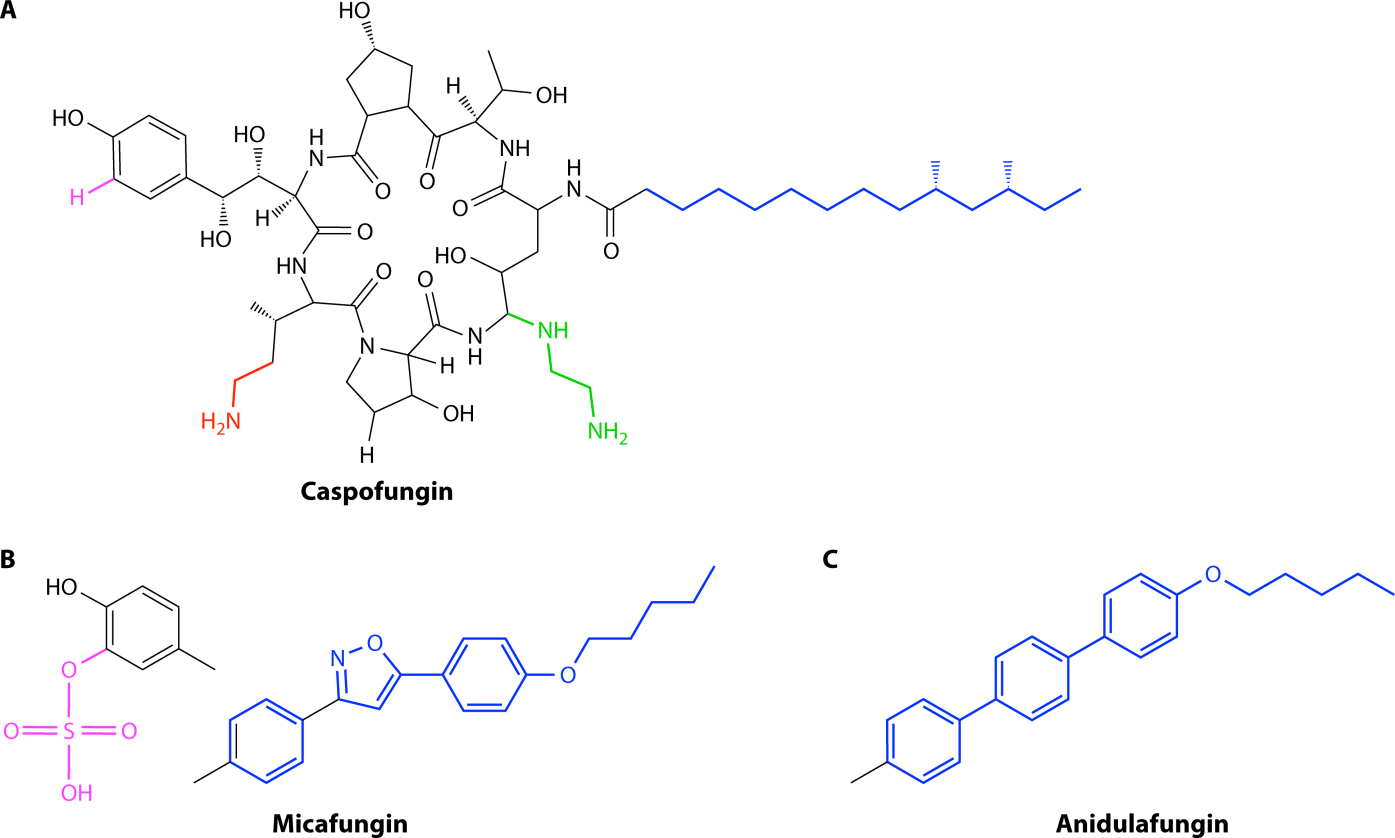
Chemical structures of representative echinocandins. Echinocandins consist of a large hexameric peptide ring with variable side chains. The antifungal drugs are modified from different echinocandin natural products, which naturally vary in side chain compositions (highlighted colors show variable chains). For caspofungin (**A**), the fatty acid chain (blue) is present in the natural precursor pneumocandin B, while the green and red side chains were synthetically added to enhance solubility and stability. (**B**) To produce micafungin, the natural precursor FR901379 was modified with the blue side chain to reduce the hemolytic activity. In contrast to caspofungin, the micafungin precursor naturally contains a sulfate group (pink) that improves water solubility. (**C**) Anidulafungin was modified from the natural precursor echinocandin B with an alcoxytriphenyl side chain (blue), reducing hemolytic properties and intercalating within the fungal membrane but with the drawback of reduced water solubility.

Due to the complexity of both the echinocandin molecules and the heteromeric target glucan synthase enzyme complex, the molecular mechanism of enzymatic inhibition is only beginning to be understood. Several molecular models and three independent cryo-electron microscopy structures of *S. cerevisiae* Fks1 have shed new light in recent years (88–90). The key to understanding the interaction between echinocandins and Fks1 likely lies in 2–3 “hotspot” regions of the protein, where mutations are frequently represented in echinocandin-resistant isolates of different species (Fig. 4). Site-directed mutagenesis and topology mapping presented a model where the hotspots were adjacent to one another and near or embedded within the outer membrane (91). This model suggested that the echinocandin peptide ring bound hotspot 1 and hotspot 2, while the lipophilic tail bound hotspot 3. The recent cryo-electron microscopy structures of Fks1 both alone and in complex with the regulatory subunit Rho1, along with molecular docking analysis, suggest that binding of echinocandin molecules at the hotspots is likely favorable (88–90, 92). Echinocandins were predicted to sterically hinder the secretion of the β-(1,3)-D-glucan polymer. Because echinocandin compounds differ in the structure of their hexapeptide cores and lipophilic tails, this model suggests slightly different binding for different drugs and, consequently, the potential for hotspot mutations to be linked to compound-specific resistance.

### Mechanisms of resistance: FKS1 mutation

As echinocandins are the preferred primary therapy for *C. auris*, the CDC has established tentative MIC breakpoints for caspofungin, micafungin, and anidulafungin. There is some evidence that caspofungin resistance may be marginally more frequent than resistance to micafungin and anidulafungin, as measured by *in vitro* assays (93). In *C. auris*, resistance mutations are reported in *FKS1* and not its paralog *FKS2*, likely connected to the former being primarily expressed in cells (94). There are rare examples of echinocandin-resistant isolates without *FKS1* mutations, but the drivers of resistance in these cases remain unknown. Two genome-wide association studies in *C. auris* have proposed non-*FKS1* candidate resistance genes, but none have been experimentally validated (95, 96).

**Fig 5:**
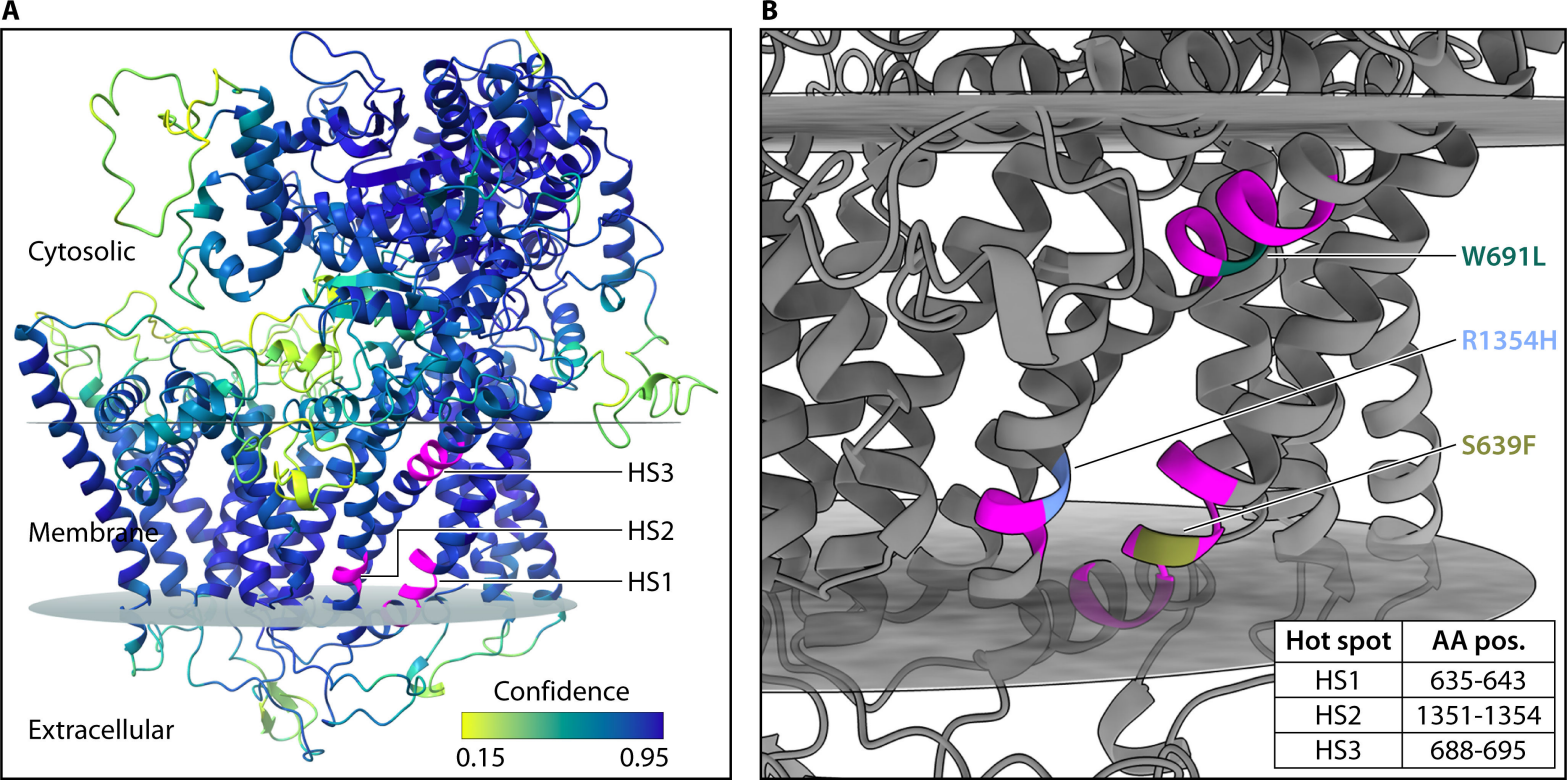
Three mutational hot spots, harboring nearly all variants linked to clinical echinocandin resistance, cluster closely together in *C. auris* Fks1. This three-dimensional structure was modeled with the SWISS-MODEL homology-modeling pipeline using a cryo-electron microscopy structure of *S. cerevisiae* Fks1 (*chimerax 1.8* [48]; PDB 8JZN). (**A**) Hot spot regions, colored magenta, were modified from (13) to reflect windows with variation among multiple medically relevant yeasts. Model confidence (QMEANDisCo [49]) was high near the three hot spots. Cell membrane boundaries (*PPM 3.0* [50]) are shown as disks. (**B**) The three hot spots, whose coordinates are shown in the table (bottom), each reside within secondary structures predicted to embed within the cell membrane. Variants experimentally validated to elevate echinocandin MICs beyond the resistance threshold are shown within their respective hot spots.

Most mutations associated with resistance are found in *FKS1* hotspot 1 (amino acid residues 634–643) in *C. auris*, while mutations in the hotspot 2 (residues 1,350–1,358) and 3 (residues 686–696) regions homologous to other species are more rarely observed (Table 1) (17, 97–99). Within hotspot 1, mutations including S639F, S639Y, S639P, F635C, F635Y, and F635Δ have been associated with clinically resistant isolates (5, 13, 17, 99–102). The most frequent among these are the mutations at position S639, comprising most echinocandin-resistant isolates (5). The homologous residue in *S. cerevisiae* Fks1 lies near the lipid-solvent interface where the protein is embedded in the membrane (Fig. 5), and a recent deep mutational scanning analysis suggests almost any mutation of this residue confers pan-echinocandin resistance (103). In line with this, experimental introduction of the S639F mutation into an echinocandin-susceptible *C. auris* isolate elevated caspofungin, micafungin, anidulafungin, and rezafungin MICs, exceeding all resistance breakpoints (104).

Within hotspot 2, mutations at R1354 have been experimentally linked to resistance. Expression of R1354H conferred microbiological resistance to anidulafungin and micafungin in a Clade I strain and to caspofungin, micafungin, and anidulafungin in a Clade III strain (94). However, there are examples of clinical isolates demonstrating susceptible micafungin MICs despite harboring this mutation (105, 106). Separately, the R1354S allele was demonstrated to confer caspofungin and anidulafungin resistance (99). In a murine infection model, *C. auris* strains carrying R1354H and R1354Y mutations were recalcitrant to micafungin and caspofungin, leading to treatment failure (15). Based on the structural models from *S. cerevisiae*, the homologous residue at this position forms part of a hydrophobic pocket that coordinates specific but differential chemical interactions with each echinocandin drug (Fig. 5) (103). Mutations at this residue in deep mutational scanning of a *S. cerevisiae FKS* gene were more likely to affect anidulafungin resistance than resistance to the other echinocandins due to a homotyrosine moiety in anidulafungin fitting within this hydrophobic pocket (103).

In hotspot 3, mutations at M690 and W691 also impact drug sensitivities. One patient isolate harboring M690V demonstrated an elevated MIC to micafungin, and a similar M690I mutation emerged during an *in vitro* evolution experiment driving caspofungin resistance (13, 106). The homologous mutation in *C. albicans* confers pan-echinocandin resistance (107). An adjacent W691L mutation was observed in a *C. auris* case in Brazil, and subsequent introduction of this mutation into an echinocandin-susceptible strain led to anidulafungin, caspofungin, and micafungin resistance (98), while the homologous mutation in *S. cerevisiae* specifically conferred resistance to caspofungin and anidulafungin (13, 98).

### Emergence of resistance upon therapy

Where echinocandin resistance remains rare in *C. auris*, the major threat of resistance is that the primary therapeutic option may wane in efficacy if resistance rates increase. As *C. auris* cases rise, reports suggest resistance to first-line echinocandins is being detected more frequently (4, 108, 109). Paired with high rates of resistance to other drug classes, echinocandin resistance threatens multi-drug or pan-resistance (2, 17, 42, 75, 110). Although rare, echinocandin-resistant isolates and pan-resistant isolates have been identified in infected individuals without prior echinocandin exposure, suggesting pan-resistant isolates can maintain transmissibility (75). Consistent with these reports, *in vitro* and murine infection data suggest resistance mechanisms in *C. auris* are not necessarily associated with apparent fitness or colonization deficiencies (14, 110, 111).

The increasing trends of echinocandin resistance from largely susceptible circulating populations suggest selection under therapy is driving resistance acquisition. Numerous case studies and outbreak investigations have reported isolation of echinocandin-resistant cultures following echinocandin treatment, often closely linking the resistant isolates to susceptible progenitors from before treatment based on phylogenetic and variant data (17, 106, 112–115). The establishment of resistance in these reports is exclusively associated with *FKS1* mutations.

The establishment of echinocandin resistance in patients under therapy can be challenging to predict. In some cases, resistant isolates are recovered days or weeks after the onset of echinocandin therapy (17, 106, 115), while other reports document the emergence of resistance years into outbreaks or even in individual patients with over a year of continual echinocandin exposure (113, 114, 116). Resistant isolates can be recovered from different infection sites after therapy even after effective clearance from other body sites, and multiple resistant isolates with different *FKS1* mutations have been recovered from the same patient. These data suggest infected patients may harbor a strong selective environment for the development of resistance (106, 112). Curiously, there are examples of resistance that appear to stem from urinary colonization (106, 117). Urinary concentrations of echinocandins are typically up to hundreds of times lower than serum concentrations (118, 119). Potentially, exposure to a limited concentration in a urinary reservoir could provide an optimal selective environment for the evolution of resistance. Where the urinary tract is among the most frequent isolation sites for *C. auris* (120), understanding the colonization status of patients may be an important consideration when monitoring the development of resistance under echinocandin therapy.

## POLYENE RESISTANCE

Resistance to the polyenes has been reported to be high in *C. auris* based on tentative breakpoints set by the CDC (4, 5, 109, 121). However, only a few isolates display high-level polyene resistance, and these have been associated with changes in the drug target ergosterol pathway (122, 123). For most *C. auris* isolates with sensitivities just above the breakpoint, there is not currently a clear molecular mechanism. To consider the variation reported in resistance rates, we first provide background on how this drug class works selectively in fungal pathogens.

### Drug background and mode of action

**Fig 6:**
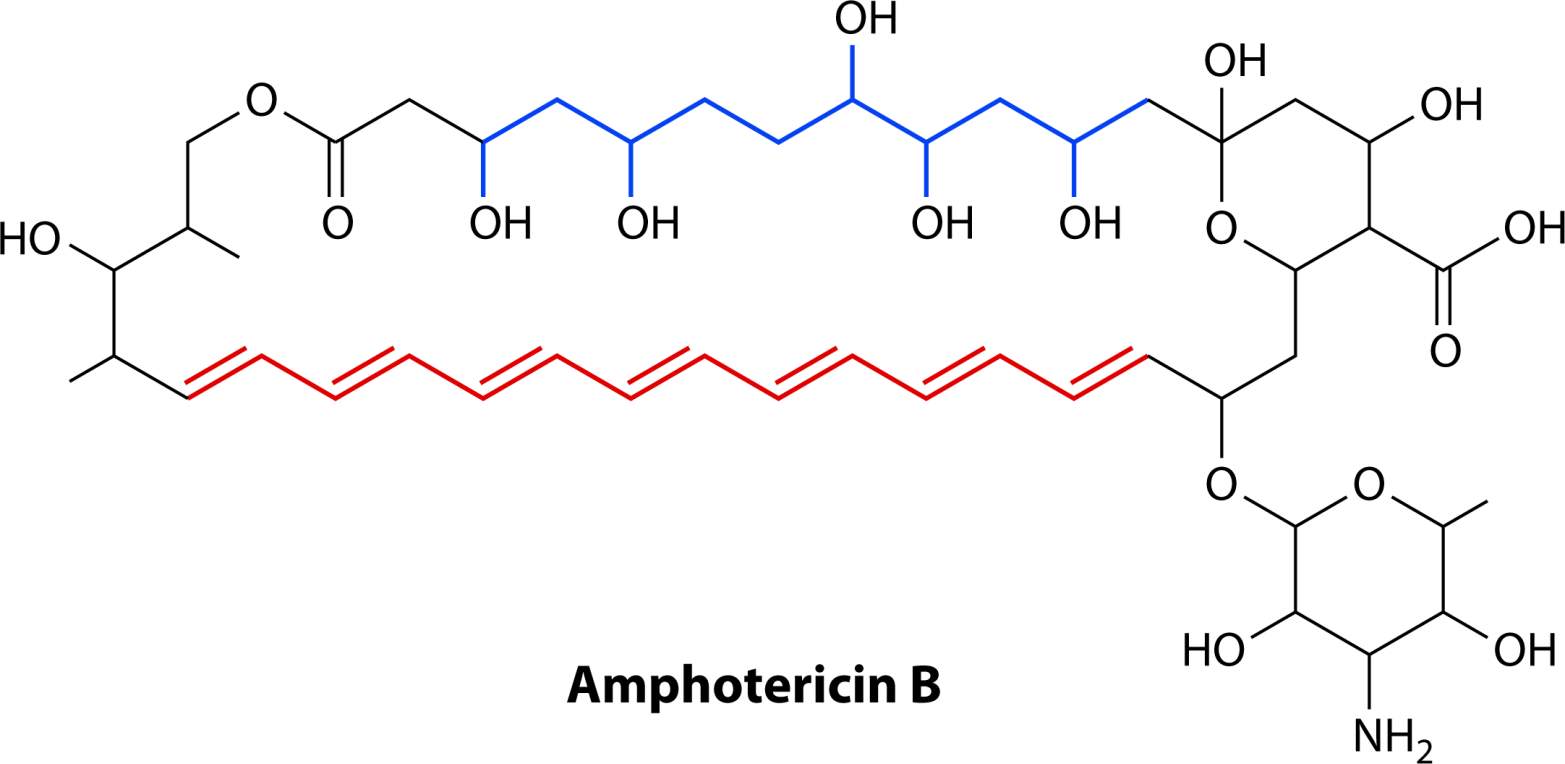
Chemical structure of amphotericin B. Polyene antifungals, such as amphotericin B, comprise both a hydrophilic polyol chain (blue) and a hydrophobic polyene chain (red). The free hydroxyl groups and the heptane chain in the drug functionally provide detergent-like properties that are critical for its membrane-active antifungal activity.

The polyenes are a class of naturally occurring macrolide drugs originally isolated as fermentation products from Actinomycete bacteria in the mid-twentieth century (124). Unlike azoles and echinocandins, polyenes are fungicidal toward *C. auris* (78). Polyene molecules consist of a hydrophilic polyol head and a hydrophobic macrolactone “polyene” tail, providing them with functional detergent-like properties (Fig. 6) (125). Unlike antibacterial macrolides such as erythromycin, the macrolactone ring in polyene antifungals contains a series of conjugated double bonds, and polyenes are virtually inactive against bacteria with completely distinct antifungal mechanisms (125).

The antifungal activity of polyenes is thought to be driven by their direct interaction with ergosterol, rather than a fungal enzyme, as in the azoles and echinocandins (126, 127). The association between polyenes and ergosterol is driven by distinct Van der Waals, hydrogen bonding, and π-π interactions at different points in the polyene molecule when conformationally aligned (127, 128). The specificity of these interactions may partly explain the selectivity of polyenes for fungal ergosterol over mammalian cholesterol. The two primary models that argue the mechanism by which polyene-ergosterol interactions lead to cell death are the pore-forming and sterol sponge models (129, 130).

In the pore-forming model, barrel-like structures comprised of polyenes and sterol molecules form transient ion channels spanning the fungal membrane (131–133). The resulting pores cause an electrolyte imbalance, ultimately resulting in cell death (131–133). Notably, high concentrations of polyenes can form similar pores even in membranes without ergosterol, suggesting the pore-forming model may not directly require binding of the polyene to the sterols (134, 135). Rather, the presence of sterols in pores is thought to enhance pore stability and dwell time (136, 137). Some refinements of this model provide a hypothesis for fungal membrane selectivity, with ergosterol promoting the development of larger and more stable channels than mammalian cholesterol (129, 136, 137).

In the sterol sponge model, polyene molecules adsorb to the membrane surface in aggregates (138–141). These clumps draw out and sequester ergosterol from the membrane (138–141). The reduced binding affinity of the polyene to cholesterol compared to ergosterol in this case suggests another mechanism of fungal selectivity (142).

There is increasing evidence for indirect mechanisms of antifungal efficacy as well. Polyene exposure has been widely observed to generate reactive oxygen species and promote oxidative damage and apoptotic-like cell death in fungi (143–146). Some reports suggest polyene resistance can be driven by antioxidant and oxidative stress response pathways (147–149).

Several natural polyenes have been isolated with formulations optimized for clinical use, but ultimately, the tetraene amphotericin B demonstrated the highest antifungal activity and has seen the widest clinical application for systemic mycoses (125). Amphotericin B is poorly water soluble, but the longest-running therapeutic formulation incorporates the compound into a sodium deoxycholate micellar suspension that can achieve high blood concentrations when administered intravenously (150). The primary drawback with amphotericin B deoxycholate therapy is associated host toxicity, the most serious manifestation being nephrotoxicity resulting in acute kidney injury in up to 50% of recipients (151, 152).

Three main lipid formulations of amphotericin B have been developed and approved for human use that show considerably less nephrotoxicity, although these are more expensive, and the impact of the lipid formulations on pharmacokinetics is less well understood (153, 154). Nevertheless, current clinical recommendations hold that lipid formulations are similarly efficacious against candidiasis as deoxycholate preparations except in urinary tract infections, due to reduced renal excretion (38). The US CDC recommends amphotericin B deoxycholate as a primary therapy for *C. auris* infection in infants too young for echinocandins, with the option to consider the liposomal formulation (https://www.cdc.gov/candida-auris/hcp/clinical-care/index.html), and as a second-stage treatment for patients who fail to respond to echinocandins.

### Mechanisms of resistance

Perhaps because of the fitness costs potentially associated with modulating ergosterol biosynthesis, polyene resistance is infrequent in *Candida* (155, 156). *C. auris* isolates tend to demonstrate elevated amphotericin B MIC values compared to many *Candida* species. This is consistent with the phylogenetically similar *Candida haemulonii* complex, members of which tend to display intrinsically reduced susceptibility to amphotericin B. This property has been proposed to derive from distinct membrane sterol content as opposed to model *Candida* species and from altered mitochondrial responses and redox homeostasis under amphotericin B stress (157, 158).

Methodological considerations may also contribute to elevated rates of amphotericin B in *C. auris* relative to other *Candida*. One explanation is that some studies report resistance rates based on the lower MIC breakpoint previously suggested by EUCAST, which was 1.0 mg/L and based primarily on evolutionary relatedness of *C. auris* to *C. albicans* (153–155). To address this gap in their recommendations, EUCAST curated a data set that corrects for clonal trends and reported that the global prevalence of amphotericin B resistance at an MIC breakpoint of 2.0 mg/L more closely resembles rates in other *Candida* species (121). A second explanation has been proposed that susceptibility testing for amphotericin B by gradient diffusion strips may yield higher MICs than by broth microdilution methods (159).

Complicating the matter, reports on microbiological resistance to amphotericin B vary considerably. One study reported 26% resistance among 2,763 *C. auris* isolates collected in the United States between 2018 and 2020 (4). Similar surveys estimated 35% resistance among early isolates from India, Pakistan, South Africa, and Venezuela (6), 50% resistance among a collection of Canadian isolates (74), and as high as 64% resistance in a northern region of Colombia (160) or 79% in a collection of isolates from Qatar (42). This regional variation makes estimating the prevalence of resistance on a global scale challenging.

Whether the naturally elevated amphotericin B MICs in *C. auris* represent a widespread acquired mode of resistance or an intrinsic reduced susceptibility, as suggested in the *C. haemulonii* complex, is not entirely clear. Acquired amphotericin B resistance in other *Candida* species often involves mutations in ergosterol biosynthesis genes *ERG2* (161, 162) and *ERG6* or combined mutations in *ERG11* with *ERG3* or *ERG5* (163, 164). Similar mutations have been reported in specific resistant *C. auris* isolates but rarely in global isolate collections, consistent with the rarity of high-level resistance in the population. In one report, subsequent to a 2-week course of amphotericin B treatment, an isolate was recovered encoding a YY98V* frameshift mutation in the *ERG6* gene and demonstrating a >32-fold increase in drug resistance compared to a matched susceptible isolate from the same patient (123). Experimental introduction of this mutation into a susceptible strain conferred a similar increase in resistance and resulted in a complete absence of ergosterol in the membrane (123). A similar case recovered an isolate with an approximately 4-fold increase in amphotericin B resistance associated with a frameshift mutation in *ERG3* and a premature stop codon in *ERG4* (122). The resistant isolate demonstrated an abolishment of ergosterol production and an accompanying growth defect. Genetic reconstitution of the wild-type alleles into this strain restored the susceptible phenotype (122).

Some ergosterol biosynthesis mutations reported in resistant clinical isolates or *in vitro-*evolved strains do not affect susceptibility. One study reported missense mutations in *ERG3* and *ERG2* from resistant clinical populations, but subsequent work identified the same alleles in drug-susceptible isolates (14, 165). *In vitro* evolution studies have identified other mutations in ergosterol biosynthesis genes, but these are often associated with dramatic fitness defects (12, 14). These reports suggest that acquired amphotericin B resistance, especially at high levels, is likely to be selectively disadvantageous, explaining its scarcity relative to the modal, lower sensitivity of the global population.

## FLUCYTOSINE RESISTANCE

Resistance to flucytosine, a pyrimidine analog rarely utilized for *C. auris* treatment, has not been as widely studied but has been documented at low levels similar to other *Candida* species. As resistance can be acquired rapidly (17, 166), flucytosine is typically in combination with other antifungals. For *C. auris*, this drug may represent an alternative therapy when other options are exhausted due to resistance or non-responsiveness to other drug classes.

### Drug background and mode of action

Flucytosine is broadly effective against most *Candida* species but is generally recommended only for application in body sites that are pharmacologically challenging for other classes of antifungals, such as the central nervous system, eye, or urine infections (38). Two major limitations preclude the wider application of flucytosine as primary therapy. First, when administered as a monotherapy, flucytosine resistance emerges at a high rate (167). Second, especially in patients with diminished renal function, high serum levels of the drug result in bone marrow toxicity, requiring careful monitoring of dosage (168). As a pyrimidine analog, the fungal metabolism of flucytosine through the pyrimidine salvage pathway usually results in the accumulation of cytotoxic metabolites (169). Genes in this pathway generally carry the mutational signatures of resistance: *FCY2* encodes the cytosine permease that imports flucytosine into the cell (170, 171), while *FCY1* encodes a fungal-specific cytosine deaminase not found in human cells (172), and *FUR1* encodes a uracil phosphoribosyl transferase (173, 174), which together process flucytosine into the toxic 5-fluoro-deoxy-UMP (175). Loss of function or missense mutations in any of these genes abolish the cytotoxic activity of flucytosine, and these mutations are readily tolerated as the fungal cells can bypass the pyrimidine salvage pathway through alternative metabolic routes (176).

In combination therapy with other classes of antifungals, the addition of flucytosine represents a viable therapeutic option for otherwise refractory *Candida* infections driven by drug-resistant *Candida* (38). Consistent with this guidance, at least one report demonstrated successful clearance of *C. auris* from the CSF of a patient following treatment with amphotericin B and flucytosine (177). Furthermore, the combination of flucytosine with any other class of antifungal has been shown *in vitro* to be microbiologically effective against otherwise pan-resistant *C. auris* isolates (178).

Since resistance breakpoints for flucytosine MIC tests have not been proposed by the CDC or EUCAST, it is challenging to estimate how common resistance is in global *C. auris* populations. Breakpoints from other *Candida* species classify roughly 14% of *C. auris* isolates as resistant (40, 179, 180). EUCAST determined an epidemiological cutoff value of 0.5 mg/L, above which they recommend considering an isolate may have acquired resistance (121).

### Mechanisms of resistance

Pyrimidine salvage pathway mutations are the most common routes to flucytosine resistance in *C. auris*. Experimental evolution found most resistant mutants harbored one of several inactivating or missense mutations in *FUR1* or *FCY2* (166). In a few cases, similar mutations have been reported to develop under flucytosine therapy in clinical isolates and were associated with elevated flucytosine MICs. One case detailed the isolation of a single flucytosine-resistant isolate recovered from a patient following the completion of a 2-week combined regimen of amphotericin B and flucytosine (11). Whole genome sequencing identified the F211I mutation in *FUR1* in this isolate (11). Another case recovered flucytosine-resistant isolates beginning 2 weeks after adding flucytosine therapy to a patient infected with a previously susceptible strain (17). In this case, the isolates of the resistant lineage harbored a 1Δ33 deletion in *FUR1* and demonstrated the absence of a detectable *FUR1* transcript by quantitative PCR (17). These examples demonstrate the emergence of *C. auris* flucytosine resistance upon therapy, further limiting potential treatment options for refractory infections. However, case data around flucytosine use against *C. auris* remain sparse, and in the absence of larger data sets, flucytosine potentially represents a viable combination therapy or treatment of last resort for combating otherwise resistant *C. auris* infections.

## DISCUSSION

*C. auris* is often referred to as a multidrug-resistant pathogen, highlighting the urgency with which this organism threatens the already limited repertoire of antifungal therapeutic options. This nomenclature is prone to misinterpretation to conflate the burden of antifungal resistance in *C. auris* into a single, insurmountable problem. In practice, *C. auris* drug resistance involves a series of distinct mechanisms and evolutionary patterns. Given limited antifungal options, it is critical to examine the details of each drug resistance mechanism independently to identify research priorities and tailor new therapeutic options.

The pattern of fluconazole resistance in *C. auris* is a distinct evolutionary story from resistance to other classes of drugs. *C. auris* demonstrates the highest prevalence of acquired fluconazole resistance among *Candida* species, such that triazoles have only limited, susceptibility-dependent use when considering therapeutic options for *C. auris* infection (181). This resistance is largely associated with mutations in the drug target *ERG11* and in transcriptional regulators of drug efflux pumps. Distinct variants conferring reduced triazole susceptibility appear to have arisen independently in at least four major clades or subclades, consistent with parallel evolution under selective pressure. With limited data on nonclinical reservoirs for *C. auris*, the original source and timing of these resistance-associated alleles remain uncertain. Nonetheless, the persistence of many of these alleles in circulating populations suggests that any associated fitness costs are minimal or offset under current ecological or therapeutic conditions. Despite the high rates of resistance, it may be valuable to better understand the clinical circumstances where triazoles could still find application against *C. auris*.

One active area of basic research exploration has been screens for additional drugs that can be used in combination with azoles (182). Natural products, repurposed drugs, and other small molecules show promise in synergistic interactions with azoles, even against resistant *Candida* strains by antagonizing drug efflux or ergosterol biosynthesis or by manipulating membrane homeostasis or cellular stress response. Current clinical guidelines do not include recommendations for combinatorial therapy with azoles, however, marking the need for further efforts into drug development and *in vivo* and clinical trial data to support the application of combinatorial therapies to overcome resistance (38, 39).

We also find compelling the possibility of other triazoles maintaining efficacy even against fluconazole-resistant *C. auris* strains. While experimental data suggest that *CDR1*-driven mechanisms confer pan-azole resistance, *MRR1*-driven mechanisms and certain *ERG11* mutations show biased resistance only to fluconazole and voriconazole. Isavuconazole and itraconazole are less recommended and less studied in the context of candidemia (39) but may represent effective therapeutic options in cases where no alternative is available because of these types of resistance mutations. The current literature profiling triazole resistance trends in *C. auris* is overwhelmingly weighted towards fluconazole; we suggest the need for large-scale profiling of the interactions between resistance mutations and alternative triazoles both *in vivo* and *in vitro*. Improvements in the understanding of structural mechanisms of drug binding, especially in pathogenic *ERG11* variants, are also likely to strengthen this line of reasoning.

By contrast, we suggest that circulating levels of acquired resistance to echinocandins, flucytosine, and possibly even polyenes are low and primarily emerge upon therapy. Re-examination of amphotericin B susceptibility testing suggests the widespread observations of unusually high resistance rates among *C. auris* may be inflated by variability in testing methods and prior placement of the tentative resistance breakpoint so near the modal MIC of circulating populations. This does suggest that *C. auris* exhibits perhaps intrinsically reduced amphotericin B susceptibility compared to common *Candida* species, and there is room to explore the biological basis driving this phenomenon. However, high-level resistance driven by acquired resistance mutations appears to be rare and comes with a substantial fitness cost. We therefore emphasize the importance of mechanistically separating intrinsic resistance mechanisms from acquired mechanisms. Limited evidence from experimental pharmacokinetic studies suggests that elevated amphotericin B dosage may be able to achieve clinical clearance even against isolates with MICs at or slightly exceeding the current tentative breakpoints. Accordingly, a recent revision to the EUCAST susceptibility testing guidelines recommends taking advantage of this fact in cases without alternative options (121). While this idea is promising, clinical and pharmacokinetic data remain sparse, highlighting the need for more comprehensive *in vivo* and clinical trial data.

Where echinocandins generally represent the primary therapy for *C. auris* infection in most regions, the threat of acquired resistance spreading is substantial. As case counts continue to rise, we emphasize the importance of public health surveillance and whole genome sequencing to monitor and trace the emergence of echinocandin resistance. Similar to the relationship between triazoles and Erg11, as well as other mutations, the possibility of different echinocandins binding Fks1 differentially under distinct resistance mutations also offers a tantalizing area for further experimental study. Growing evidence from *in vitro*, biochemical, and structural modeling studies suggests that certain Fks1 mutations do not confer universal resistance to echinocandins. Refinement of these models and, more importantly, application to *in vivo* and clinical use cases may ultimately provide a framework for a tailored echinocandin therapy even against strains harboring Fks1 resistance mutations. The impact of such circulating mutations in resistance to Ibrexafungerp, which also inhibits β-(1,3)-D-glucan synthesis, will also become more important as its adoption as an echinocandin alternative progresses.

With the continued rise in clinical cases of *C. auris*, there is a well-established urgency to refine understanding of resistance mechanisms, monitor the emergence of resistance in circulating populations, and examine the patterns where resistance emerges during antifungal therapy.
